# Specific LTR-Retrotransposons Show Copy Number Variations between Wild and Cultivated Sunflowers

**DOI:** 10.3390/genes9090433

**Published:** 2018-08-29

**Authors:** Flavia Mascagni, Alberto Vangelisti, Tommaso Giordani, Andrea Cavallini, Lucia Natali

**Affiliations:** Department of Agricultural, Food, and Environmental Sciences, University of Pisa, Via del Borghetto 80, I-56124 Pisa, Italy; flavia.mascagni@unipi.it (F.M.); albertovangelisti@libero.it (A.V.); tommaso.giordani@unipi.it (T.G.)

**Keywords:** *Helianthus annuus*, long terminal repeat retrotransposons, plant domestication, retrotransposon abundance, retrotransposon proximity to genes

## Abstract

The relationship between variation of the repetitive component of the genome and domestication in plant species is not fully understood. In previous work, variations in the abundance and proximity to genes of long terminal repeats (LTR)-retrotransposons of sunflower (*Helianthus annuus* L.) were investigated by Illumina DNA sequencingtocompare cultivars and wild accessions. In this study, we annotated and characterized 22 specific retrotransposon families whose abundance varies between domesticated and wild genotypes. These families mostly belonged to the Chromovirus lineage of the Gypsy superfamily and were distributed overall chromosomes. They were also analyzed in respect to their proximity to genes. Genes close to retrotransposon were classified according to biochemical pathways, and differences between domesticated and wild genotypes are shown. These data suggest that structural variations related to retrotransposons might have occurred to produce phenotypic variation between wild and domesticated genotypes, possibly by affecting the expression of genes that lie close to inserted or deleted retrotransposons and belong to specific biochemical pathways as those involved in plant stress responses.

## 1. Introduction

Transposable elements (TEs) are DNA sequences that are able to change their position in the chromosomes. They are classified into two classes depending on whether the transposition intermediate is RNA (Class I transposons or retrotransposons) or DNA (Class II or DNA transposons [1]. Class I elements are found in most eukaryotic lineages. In plants, the most abundant retrotransposon order is that with long terminal repeats (LTRs), two direct repeats containing promoter and RNA processing signals, flanking a region encoding a polyprotein that includes the enzymes necessary for its transposition [1]. Plant LTR-retrotransposons (LTR-REs) are classified into two main superfamilies—Copia and Gypsy [1]—which differ in the order of the enzymes within the polyprotein [2]. LTR-RE length ranges from a few hundred base pairs to over 10 kbp [2]. Copia and Gypsy superfamilies are in turn classified into different major lineages based on sequence similarity [3,4,5]. However, DNA sequence similarity within a lineage is minimal and limited to coding regions. When sequence similarity within a lineage extends to noncoding portions, two elements may be grouped in a single family [1].

Retrotransposons transpose by producing an RNA intermediate that is then reverse transcribed to DNA and inserted at a new genome site [2]. This transposition mechanism, which uses enzymes produced by the retrotransposon itself, a reverse transcriptase, a ribonuclease (RNAase), a protease, and an integrase, implies the production of a new copy for each transposition event. Retrotransposon mobility in the genome is usually blocked by different epigenetic mechanisms; however, some retrotransposons under certain environmental conditions are able to escape epigenetic control by the host genome [6]. This escape can result in a huge increase in retrotransposon copy number and consequently an extremely rapid and large increase in genome size when one considers evolutionary timescales [1].

Even so, the retrotransposon component of the eukaryotic genomes is subject to rapid turnover [7,8]. While retrotransposons can increase in number in a relatively short time span, they can also be rapidly removed from the genome through the processes of unequal homologous and illegitimate recombination [9,10].

Retrotransposons proliferation and loss can lead to the creation of haplotypes with different LTR-RE numbers at specific loci [11,12]. Hence, the number of LTR-REs in a genome can also change because of random combination between LTR-RE-rich or poor haplotypes.

Retrotransposon activity produces genetic variation with important effects on the evolution of a species [13]. Transposons may insert in or near a gene, resulting in direct alteration of the coding sequence, transcription regulation modification, or altered splicing patterns [14]. Insertion in the proximity to a gene may also have consequences; since retrotransposons are epigenetically inactivated by the host, integration of an element may actually modify the epigenetic setting of the insertion site. Overall, retrotransposons are known to regulate the epigenetic setting of the genome and chromatin organization and structure. Perhaps more importantly, insertion or removal of a retrotransposon can change the expression rate or regulation of neighboring genes [15,16,17,18].

A few studies have investigated the possible role of transposable elements and other repetitive elements of genomes in the domestication of crop plants [19] and have included work on maize [20,21], rice [22], and sunflower [23].

The sunflower (*Helianthus annuus* L., Asteraceae) is one of the most important oilseed crops. The origin of the genus *Helianthus* dates back 4.75–22.7 million years [24]. It is likely the sunflower originated in Mexico and then spread through North America [25]. The first domestication of sunflower probably occurred in the eastern regions of North America. Although archaeological studies argued for an earlier cultivation in Mexico [26], molecular genetic studies have shown that modern sunflower cultivars are most genetically similar to wild accessions of the Midwestern USA [27,28]. Thus, it appears that sunflower was domesticated by Native Americans in eastern North America. The early domesticated genotypes were introduced to Europe at the beginning of the 16th century by naturalists [29,30,31]. A massive breeding program for high oil yield developed in Russia in the 19th century. In fact, even in North America, the first widespread cultivars were derived from materials reintroduced from Russia from this breeding program [32,33,34]. This implies a strongly reduced genetic variability in cultivated sunflowers in comparison to wild accessions, which colonized and adapted to multiple different environments [35].

Indeed, modern sunflower cultivars are quite different from wild accessions. They are generally single-headed, have specific oil profiles, and are dwarf. By the early 1970s, a massive increase in hybrid seed production occurred due to the availability of different heterotic groups of inbred lines as well as a system of cytoplasmic male sterility and fertility restoration derived from interspecific crosses with *Helianthus petiolaris* [36].

A number of studies have shown that genes affecting branching and other features of plant architecture, fatty acid biosynthesis, and flowering time were involved in sunflower domestication [37,38,39,40,41,42]. Baute et al. [41] analyzed the transcriptomes of wild and cultivated sunflowers and identified 137 genes associated with domestication and improvement, as indicated by their low sequence variability in domesticated genotypes compared to wild accessions. As in the previous studies, genes putatively involved in fatty acid biosynthesis, as well as in branching, were largely represented.

More recently, other authors [43] analyzing transcriptomes of wild and domesticated sunflowers have identified differential splicing divergence related to domestication, especially through intron retention. Differential splicing has been related to genes involved in functions related to seed development. Many differential splicing patterns in cultivars probably derived from wild accessions, increasing their frequency because of selection during domestication.

The involvement of variation in the repetitive component, and especially of retrotransposon copy number, in sunflower domestication was first studied by Mascagni et al. [23]. The sunflower has a large genome of about 3.6 Gbp [42]. Its repetitive component accounts for around 80% of the genome and is mostly composed of LTR-REs [44,45,46,47,48], especially of the Gypsy superfamily and Chromovirus lineage. High levels of LTR-RE-related polymorphism have been found in both wild and cultivated genotypes [49].

Mobilization and consequent changes in the abundance of retrotransposons have occurred during *Helianthus* speciation, even in relatively recent times [50,51]. Sunflower LTR-REs are apparently transcribed and, although at low rates, reinserted into the genome, even in nonstressful environmental conditions [52].

In a previous study [23], a library of 123 LTR-retrotransposon sequence families of sunflower was produced assembling a set of 454 sequence reads of the HA412-HO line using RepeatExplorer (https://galaxy-elixir.cerit-sc.cz), a repetitive sequence online clustering tool [53]. Each cluster represents an individual family of repetitive elements, which show large sequence similarity and share a common progenitor [53]. Mascagni et al. [23] identified clusters belonging to the Gypsy and the Copia superfamilies (85 and 38 sequence families, respectively). The lineage (indicated as family in that work) of each cluster was also identified. Different clusters belonging to the same LTR-RE lineage can be defined as different LTR-RE families of that lineage. Mascagni et al. [23] showed changes in the abundance of certain lineages of Gypsy and Copia LTR-REs between cultivated and wild genotypes of sunflower. Moreover, they found differences in LTR-RE number lying proximal to gene coding sequences among the same genotypes.

Here, we extend the previous study [23], performing a new comparative analysis of LTR-REs between wild and domesticated genotypes of *H. annuus* at the family level in order to identify the involvement of specific LTR-RE families in retrotransposon-related structural variations and how they define cultivars in comparison to wild plants. Moreover, an analysis of the chromosomal localization of these LTR-RE families and of their supposed association to gene coding sequences was conducted, allowing us to hypothesize on the role of such structural variations in the domestication of the sunflower.

## 2. Materials and Methods 

### 2.1. Plant Genotypes and Illumina Sequences Used in the Analyses

The sunflower cultivars and wild accessions used in this study were the same used by Mascagni et al. [23] (Table 1). We selected 7 wild accessions of *H. annuus* from different regions of North America, and 8 cultivars randomly selected from different countries in which sunflower seeds are massively produced, one cultivar per country. Wild accessions and cultivars were obtained from the United State Department of Agriculture, Agricultural Research Service (USDA-ARS), National Genetic Resources Program, USA. Further data on the genotypes can be found at the US National Plant Germplasm System webpage (http://www.ars-grin.gov/npgs/searchgrin.html) and in previous studies of ours [23,54]. 

Raw Illumina paired-end sequences from DNA isolated from leaves of single individuals of each genotype were available at the Sequence Read Archive (SRA) of NCBI (BioProject number PRJNA302358). Illumina reads were preprocessed [23] to remove Illumina adapters, then quality-trimmed with default settings, and the lengths of reads were defined at 90 nt.

### 2.2. Long Terminal Repeats-Retrotransposon Redundancy Estimation

A reference library of 11,546 contigs, belonging to 123 LTR-REs families and representative of all sunflower LTR-Res, was available [23]. This was obtained by graph-based clustering of sequences of the highly inbred sunflower line HA412-HO using RepeatExplorer [53].

This library was used as reference for mapping Illumina reads of each genotype. Mapping was carried out using an updated version of CLC-BIO Genomic Workbench (version 9.5.3, CLC-BIO, Aahrus, Denmark), with the following parameters: mismatch cost = 1, deletion cost = 1, insertion cost = 1, similarity = 0.9, and length fraction = 0.9.

Using this tool, those reads that match multiple distinct sequences were distributed randomly and, hence, the number of reads that matched to a single sequence simply gave an indication of its abundance. However, if all sequences of a sequence family (i.e., sequences that shared sufficient similarity to form a cluster) were taken together, the total number of mapped reads for that cluster (compared to the total number of all genomic reads) indicated the effective abundance of that family. Abundance values were reported as total number of mapped reads per million reads used for mapping.

The occurrence of retrotransposon abundance variation among wild and domesticated genotypes was estimated by a principal component analysis (PCA) and a permutational multivariate analysis of variance (PERMANOVA) [55]. For each family, the abundance data on 15 genotypes were used to build a Euclidean distance matrix. PCA was performed by implementation of R package FactoMineR version 1.26 [56]; PERMANOVA used R package vegan version 2.0-10 [57]. An in-house R script was used for performing statistical tests for all the families. Differences between wild and cultivated genotypes were considered significant when *p* ≤ 0.01.

### 2.3. Retrotransposon Distribution along the Sunflower (HanXRQInbred Line) Genome

Using RepeatMasker (http://www.repeatmasker.org), each of the 17 linkage groups (LGs) of the only currently available sunflower genome sequence—the HanXRQ inbred line [42]—were compared with the datasets of Gypsy or Copia families, which showed significant differences in abundance between wild and cultivated genotypes. In addition, the analysis was also performed against a putative sunflower centromeric sequence—HAG002P01 [58]—separately under default parameters but -div 20. All LGs were then subdivided into 3-Mbp-long regions using an in-house Perl script. The number of masked bases was then counted for each 3 Mbp fragment using another in-house Perl script.

### 2.4. Analysis of Proximity of Long Terminal Repeats-Retrotransposons to Genes

For each genotype, a set of Illumina paired-end reads (trimmed for quality and adapters but not for specific length) was mapped onto a library containing a subset of LTR-RE families, assembled by an online clustering tool, RepeatExplorer (https://galaxy-elixir.cerit-sc.cz/), which showed differentiating abundance variation between cultivated and wild genotypes. They were also similarly mapped to a set of genes representing the whole sunflower transcriptome [59].

Mapping was performed using a Burrows–Wheeler Aligner (BWA) version 0.7.10-r789 [60] with the following parameters: aln -t 4 -l 12 -n 4 -k 2 -o 3 -e 3 -M 2 -O 6 -E 3. The resulting paired-end mappings were resolved with the “sampe” module of BWA, and the output was converted into a “bam” file using SAMtools version 0.1.19 [61]. SAMtools was used to extract the reads mapping in pairs with the function ”view”, option -F 12.

For each genotype, all read pairs where one read mapped onto a LTR-RE family and the other onto a gene sequence were selected, and the reads relating to the gene sequences were collected. Then, the corresponding gene sequences were retrieved from the HanXRQ genome annotation database (https://www.heliagene.org/HanXRQ-SUNRISE/), and Blast2GO [62] was used to identify the corresponding Kyoto encyclopedia of genes and genomes (KEGG) pathways (https://www.genome.jp/kegg/). Significant differences in the number of identified KEGG terms between cultivars and wild accessions were assessed by PERMANOVA, as described above.

## 3. Results

### 3.1. Some Long Terminal Repeats-Retrotransposon Families Show Significant Differences in Abundance between Wild and Cultivated Genotypes

An estimate of structural variation relating to the mobilization of LTR-REs can be determined by the increase or decrease of coverage by certain elements in the different genotypes [63]. In our study, the coverage of each family was determined in seven wild accessions of *H. annuus* from different regions of North America, and eight cultivars were randomly selected from different countries in which sunflower seeds are massively produced, one cultivar per country. In doing so, we were attempting to get a representative sample of diversity in the domesticated and wild gene pools of *H. annuus*. In fact, large genetic diversity among these genotypes has already been assessed using retrotransposon-based molecular markers [49]. 

The abundance of each of the 123 LTR-RE families contained in the reference library [23] in each accession was measured by counting the total number of reads (per million) that mapped onto the contigs belonging to such families. This method, which assumed that Illumina reads were sampled with uniform biases (among genotypes of the same species) for particular sequence types, if any, has been used in many species [64,65,66,67], including sunflower [23,46,54,68]. This method was previously validated by slot blot hybridization for two *Helianthus* genotypes [54]. 

**Figure 1:**
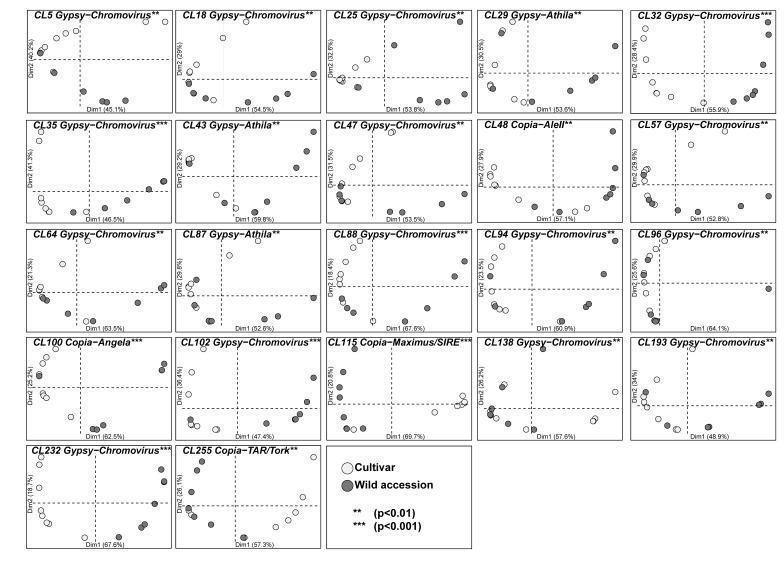
Principal component analysis (PCA) plots of abundance values of 22 LTR-RE families in domesticated (white dots) and wild genotypes (grey dots) of *Heliantus annuus*. The percentage of variation accounted by each axis is shown. Asterisks indicate permutational multivariate analysis of variance (PERMANOVA) significance between cultivars and wild accessions: *** *p* < 0.001; ** *p* < 0.01.

Principal component analysis (PCA) of the intraspecific relative abundance of each of the 123 LTR-RE families was performed. No significant abundance variation was observed for the majority of LTR-RE families (101 out of 123 families). For 22 out of 123 families, wild and cultivated genotypes showed significant differences (*p* ≤ 0.01; Figure 1). The abundance values (in millions of mapped reads per million) for each of the 22 LTR-RE families in the 15 selected genotypes are reported in Table S1. 

**Figure 2:**
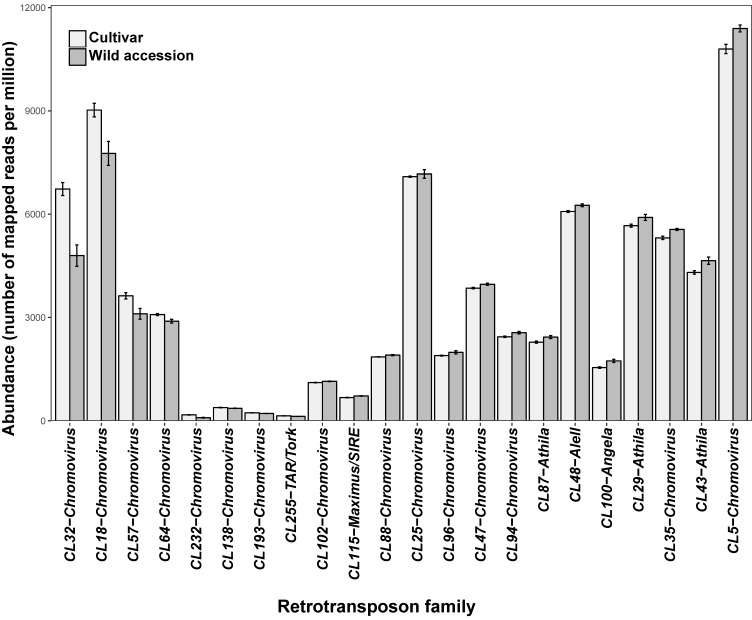
Mean number (± standard error) of mapped reads (x million) of each of the 22 LTR-RE families in eight cultivars and seven wild accessions of *H. annuus*. All families were differentially abundant by PERMANOVA, at *p* < 0.01.

**Figure 3:**
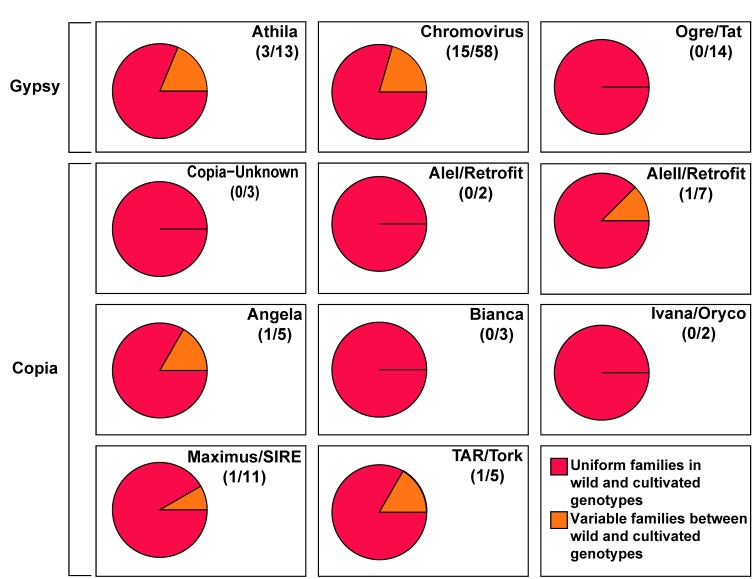
Percentage of long terminal repeats-retrotransposons (LTR-RE) families showing significant differences in abundance between wild and cultivated genotypes, according to the lineage to which such families belong. For each lineage, the total number of families in the sunflower genome is reported in parentheses.

In eight out of 22 LTR-RE families (seven of the Gypsy-Chromovirus lineage and one of the Copia-TAR/Tork lineage), the mean abundance was higher in cultivars than in wild accessions; the opposite trend was observed in the other 14 families (eight Gypsy-Chromovirus, three Gypsy-Athila, one Copia-Maximus/SIRE, one Copia-Angela, and one Copia-AleII; Figure 2). The percentage of LTR-RE families in which variation was higher in some lineages (such as Chromovirus and Athila of the Gypsy superfamily) than in other is shown in Figure 3.

### 3.2. Chromosomal Localization of Long Terminal Repeats-Retrotransposons Families

The 22 LTR-RE families showing significant variation in abundance between wild and cultivated genotypes were mapped to the only currently available genome sequence of *H. annuus* (HanXRQ inbred line [42]) using contigs from each family, though keeping the Gypsy and Copia superfamilies separated. To structurally describe the 17 linkage groups of the HanXRQ sequence, masking was also performed using a sequence that was previously described as interspersed, but with a prevalent centromeric localization by FISH [58], in order to identify putative centromeres.

**Figure 4:**
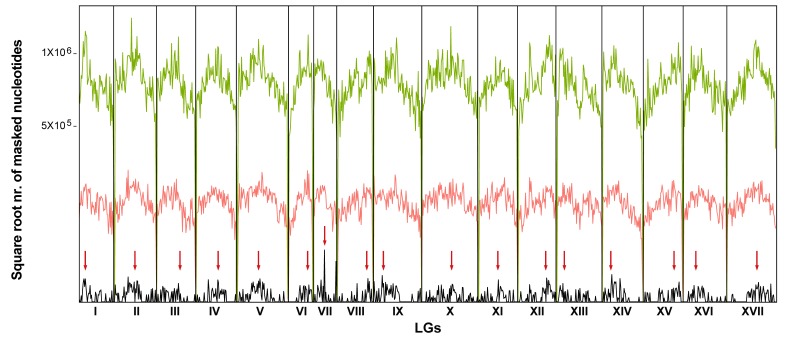
Distribution of the Gypsy (in green) and Copia (in red) LTR-RE families along the 17 linkage groups (LGs) of the sunflower genome (line HanXRQ [42]). The distribution of a putative centromeric sequence ([58], in black) is also reported. Red arrows indicate the most probable centromere position of each LG, corresponding to the peaks of highest frequency of the putative centromeric sequence. The space of each LG is proportional to its length in nucleotides.

The localization of 18 Gypsy and four Copia families is reported in Figure 4. No preferential localization was observed for Copia LTR-RE families, which were interspersed in the genome. While Gypsy families were also interspersed in the genome, similarly to Copia families, masking data suggested a preferential localization of Gypsy LTR-REs around putative centromeres in certain LGs (e.g., LGs I, IV, V, XI, XII, XV). This was consistent with the vast majority of Gypsy families that belong to the Chromovirus lineage, which has been shown to be involved in centromere structure [69].

### 3.3. Proximity of Retrotransposons to Genes

To evaluate the potential impact of LTR-RE insertions on overall gene function, we analyzed the association between sequences belonging to the 22 LTR-RE families showing significant differences in abundance between wild and cultivated genotypes and protein encoding genes in the genome.

The proximity of LTR-REs to genes in the wild accessions and in the cultivars of *H. annuus* was estimated by mapping Illumina paired-end reads to both the library of 22 differently abundant LTR-RE families and to a set of sequences representing the whole sunflower transcriptome [59]. The Illumina paired-end reads of which one mapped onto an LTR-RE and the other onto a gene (hereafter called gene-RE pairs) were retained from every accession (Table S2). Because of the relatively low number of genomic reads and to counter random variation, it was decided two groups of sequence would be created: gene-RE pairs from all wild genotypes and gene-RE pairs from all cultivated genotypes. In fact, because of the relatively low coverage of genomic reads used in this analysis for each genotype, any differences between single genotypes could have been determined by the stochasticity in read packages used for mapping. On the contrary, the effect of stochastic variation was greatly reduced by pooling all gene-RE pairs of all the wild genotypes and those of all the cultivars. The number of gene-RE pairs (per million reads) for each RE family in cultivars and wild accessions is reported in Table 2. On average, families of the Gypsy superfamily showed a higher number of gene-RE pairs per million reads in wild than in cultivated genotypes; for some families of the Chromovirus and Athila lineages, such differences were statistically significant (Table 2). By comparison, the number of gene-RE pairs per million reads did not change when considering LTR-RE families of the Copia superfamily in the wild and cultivated groupings.

The list of genes lying in proximity to elements belonging to LTR-RE families that showed different frequencies between cultivated and wild genotypes is reported in Table S3. A few of these genes have already been reported as being important during sunflower domestication [41] based on their sequence conservation in domesticated genotypes. Genes in cultivars included sequences for an iron-regulated protein 3, a protein of the EamA-like transporter family, an *O*-glycosyl hydrolase, a putative myosin, and an ATP synthase, subunit β. In wild accessions, sequences included genes for a carbon–sulfur lyase, a protein of the AMP-dependent synthetase and ligase family, a *P*-glycoprotein, a protein of the RING/U-box superfamily, and an RNA polymerase II transcription mediator.

**Figure 5:**
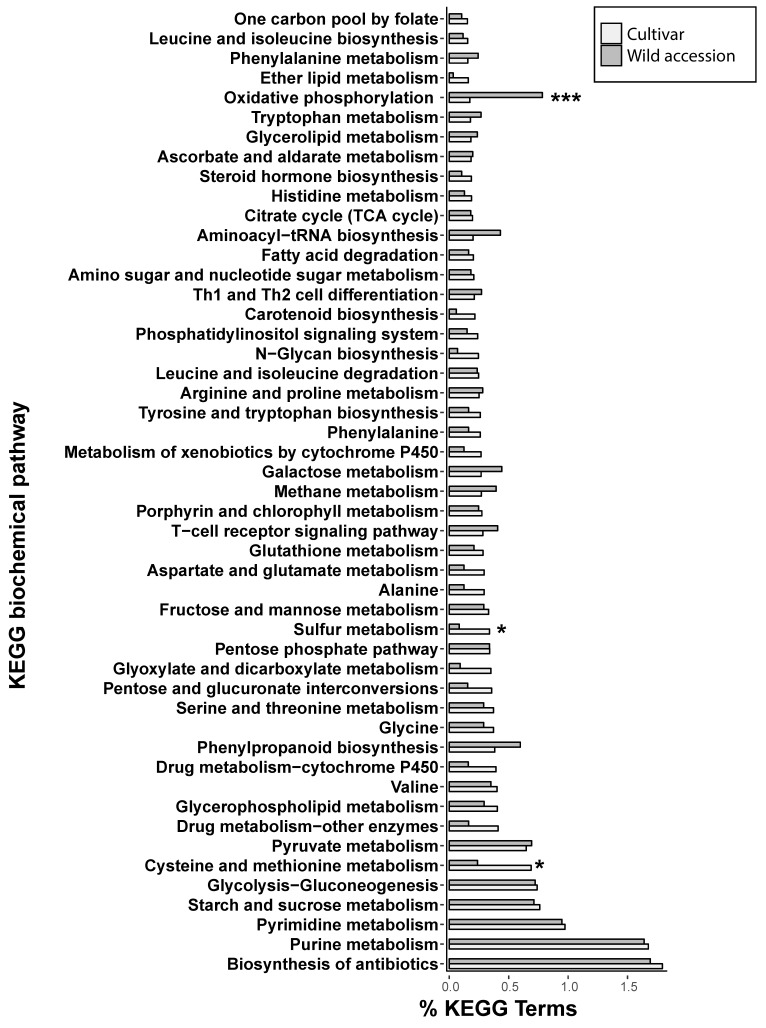
Percentages of Kyoto encyclopedia of genes and genomes (KEGG) biochemical pathway terms associated with gene-RE pairs in cultivars and wild accessions of *H. annuus*. Asterisks indicate PERMANOVA significance between cultivars and wild accessions: *** *p* < 0.001; * *p* < 0.05.

In order to evaluate how such gene products might be involved in biochemical processes such that LTR-REs insertion could induce phenotypic variation between wild and cultivated genotypes, we identified the KEGG biochemical pathways of the genes lying near these LTR-REs (Figure 5). Comparing the percentages of KEGG terms between cultivated and wild genotypes, significant differences were observed for three biochemical pathways, oxidative phosphorylation, sulfur metabolism, and cysteine and methionine metabolism (Figure 5).

## 4. Discussion

It is commonly accepted that only a few loci are involved in the process of domestication of a species from its wild progenitor [70,71,72,73]. This could imply that sequence divergence between domesticated and wild genotypes might be more pronounced in those few loci that, for example, might provide characters that are favorable in cultivation but are neutral (or even negatively selected) in the wild. In these loci, extensive molecular divergence can be observed [74]. In addition, reduction in population size during artificial selection does contribute to making domesticated and wild genotypes more divergent [75]. Based on this hypothesis, 122 genes involved in sunflower domestication and 15 genes involved in sunflower cultivar improvement were identified [41].

Although coding sequence variations (and selection of specific alleles) would have played a major role in the process of domestication, other genetic mechanisms are also likely to have played a substantial part in this process, for example, differential regulation of gene expression including processes such as alternative splicing [76]. Recently, Smith et al. [43] showed an association of alternative splicing of some genes and domestication in sunflower.

Considering that phenotypic changes may arise from changes in the regulation pattern of genes, which are often derived from variation in neighboring noncoding, cis-regulatory sequences [20,73], variations in the repetitive component related to retrotransposon insertions/deletions could have had a primary role in determining the phenotypes that were selected by humans during plant domestication.

In previous experiments [23], significant differences in abundance of certain lineages of Gypsy and Copia LTR-REs were measured between cultivated and wild genotypes of sunflower. The present study extends those findings at the retrotransposon family level, showing that 22 of 123 LTR-RE families were significantly different in abundance between cultivated and wild genotypes. In addition, within a lineage, some families were significantly more abundant in cultivars, while others were more abundant in wild accessions. The level of variation might be related to there being relatively few genotypes due to initial selection by early European explorers and the breeding program in Russia [27]. If it is assumed that the differences in LTR-RE abundance did not result in favorable traits of selected and bred genotypes, then the smaller or higher LTR-RE abundance of a specific family in cultivars than in wild accessions might be a consequence of genetic drift. However, if such changes in abundance of certain families resulted in favorable traits of those genotypes, it might be deduced that low or high abundance of certain LTR-RE families were unconsciously selected by the first breeders.

Long terminal repeats-retrotransposons families that showed different abundances between wild and cultivated plants were mapped to the sequenced inbred line of sunflower [42]. LTR-RE families were not confined to specific chromosomal regions notoriously filled with repetitive elements; rather, they were interspersed over all chromosomes. As such, structural variations related to these LTR-REs also occurred in gene-containing chromosomal regions.

Retrotransposon insertions or deletions may affect the phenotype of the host, especially when they occur in the proximity to genes whose expression rate they influence [77,78]. Retrotransposons mobilization may also affect the plant phenotype by modifying the epigenetic setting of the locus. This is because integration of a retrotransposon is generally accompanied by methylation of the insertion region, with consequent inactivation of proximal genes [17].

We assessed the occurrence of insertions/deletions in proximity to genes of the 22 LTR-REs belonging to families whose abundance changed between wild and cultivated sunflowers. Paired reads where one mapped onto an LTR-RE and the other onto a gene were identified. This indicated the proximity of LTR-RE insertions to genes they might influence. However, we could not exclude the possibility that in some cases, these gene sequences might have been unfunctional (e.g., portions of genes, pseudogenes).

For some LTR-RE families, the level of proximity to genes was significantly different between wild accessions and cultivars, most notably with families belonging to Chromovirus and Athila lineages of the Gypsy superfamily.

Interestingly, some of the genes indicated by Baute et al. [41] to be involved in sunflower domestication and improvement as determined by their sequence conservation, also showed differences in proximity to LTR-REs between cultivated and wild genotypes. Such differences could have contributed to the large phenotypic differences between wild and domesticated sunflowers. Analysis of the biochemical processes of genes with differentiating proximity to LTR-REs between wild and cultivated genotypes identified at least three important KEGG pathways: oxidative phosphorylation, sulfur metabolism, and cysteine and methionine metabolism. 

Oxidative phosphorylation is a fundamental process in energy metabolism whereby cells oxidize nutrients, releasing energy for ATP production (see for example Reference [79]).

The sulfur metabolism and related cysteine and methionine metabolism pathways also play an important role in plants as they are involved in the production of reduced sulfur compounds for the biosynthesis of S-containing amino acids. These pathways are related to oxidative phosphorylation as sulfur compound production starts from the activation of sulfate with ATP to form adenylyl sulfate [80]. Among a large range of functions, sulfur-containing defense compounds are crucial for the survival of plants under abiotic and biotic stresses [81]. During drought stress, sulfur compounds have specific roles with the biosynthesis of osmolytes and osmoprotectants, such as polyamines, and the production of glutathione and its precursor cysteine are also increased [82].

A common feature of these biochemical processes is that they participate in some way in the response of plants to abiotic and biotic stress. It is known that a common trait of domesticated plants is an increasing susceptibility to environmental stresses [83]. It is possible that retrotransposon mobility might have affected this trait during sunflower domestication.

In conclusion, our study identified LTR-RE families specifically involved in structural variations between wild and cultivated sunflower genotypes. Our data suggest that such structural variations occurred in some cases near coding genes, with possible consequences on their expression and consequently on the phenotype. In this sense, the occurrence of LTR-RE-related structural variation represents a further process that might have affected plant domestication, alongside the selection of alleles of specific genes. This study indicates what LTR-RE families and what genes should be taken into account in future studies on the importance of changes in the repetitive fraction of the genome in sunflower domestication. Resequencing the genome of some domesticated and wild sunflower genotypes will allow a precise measurement of the extent of LTR-RE-related structural variations and their localization to specific genes. Expression analysis of such genes will allow the effect of LTR-RE-related structural variation on the domesticated sunflower phenotype to be defined with more precision.

## Figures and Tables

**Table 1:** Sunflower genotypes used in this study. For each genotype, the United State Department of Agriculture (USDA) identification code, the area of cultivation for domesticated genotypes, and the number of reads sequenced by the Illumina technique are reported. Reads were trimmed at 90 nt and used in analyses as single ends for measuring LTR-retrotransposons (LTR-REs) abundance. For analyzing the proximity between LTR-REs and genes, paired ends were used and no specific length was defined.

Type	Name	Id Code	Area of Cultivation	Raw Reads	Trimmed Reads (as Single Ends, 90 nt)	Trimmed Reads (as Paired Ends)
Domesticated	Hata	Ames 22503	Argentina	32,100,390	31,085,284	31,624,960
	Dussol	Ames 22499	France	25,678,406	24,988,640	25,375,912
	Argentario	Ames 1842	Italy	10,759,866	10,134,402	10,566,652
	Karlik	Ames 3454	Spain	23,499,752	22,938,364	23,087,458
	Zelenka	Ames 22530	Russia	9,048,276	8,824,270	8,858,154
	Roman “A”	PI531386	Romania	19,408,888	18,621,244	19,095,974
	HOPI	PI369359	USA	15,768,198	15,254,502	15,437,790
	Seneca	PI369360	USA	13,911,506	13,334,732	13,667,436
Wild	Arizona (AZ)	Ames14400	-	14,641,510	14,013,588	14,357,666
	Colorado (CO)	PI586840	-	23,335,576	21,965,694	22,916,284
	Illinois (IL)	PI 435540	-	18,577,580	17,366,768	18,145,470
	Kentucky (KY)	PI 435613	-	14,853,802	13,845,748	14,580,828
	Mississippi (MS)	PI 435608	-	22,921,544	21,376,594	22,226,864
	North Dakota (ND)	PI586811	-	51,681,332	47,906,352	49,574,892
	Washington (WA)	PI 531018	-	6,996,658	6,479,624	6,724,410

**Table 2:** Mean number (per million reads) of paired reads where one read maps to a LTR-RE family and the other to a gene sequence. For each family, the lineage and the superfamily are reported. Statistical significance of differences between cultivars and wild accessions was assessed by PERMANOVA (*** *p* < 0.001; * *p* < 0.05).

Superfamily	Lineage	Family	Mean nr. of Gene-RE Mapping Paired Reads per Million Reads		
			Cultivars	Wild accessions	PERMANOVA
Gypsy	Chromovirus	CL5	2.95	4.05	
Gypsy	Chromovirus	CL18	1.61	1.64	
Gypsy	Chromovirus	CL25	4.37	6.53	*
Gypsy	Chromovirus	CL32	1.07	0.80	
Gypsy	Chromovirus	CL35	0.80	0.90	
Gypsy	Chromovirus	CL47	0.84	1.57	***
Gypsy	Chromovirus	CL57	0.73	1.04	*
Gypsy	Chromovirus	CL64	0.47	0.65	
Gypsy	Chromovirus	CL88	0.47	0.81	*
Gypsy	Chromovirus	CL94	0.25	0.32	
Gypsy	Chromovirus	CL96	0.76	0.82	
Gypsy	Chromovirus	CL102	0.14	0.18	
Gypsy	Chromovirus	CL138	0.17	0.14	
Gypsy	Chromovirus	CL193	0.03	0.05	
Gypsy	Chromovirus	CL232	0.03	0.01	
Gypsy	Athila	CL29	1.16	1.34	
Gypsy	Athila	CL43	1.31	1.90	*
Gypsy	Athila	CL87	0.42	0.64	*
Mean Gypsy			0.98	1.38	
Copia	AleII	CL48	1.08	1.08	
Copia	Maximus/SIRE	CL115	0.12	0.21	
Copia	Angela	CL100	0.27	0.29	
Copia	TAR/Tork	CL255	0.18	0.17	
Mean Copia			0.41	0.44	

## Supplementary Materials

The following are available online at http://www.mdpi.com/2073-4425/9/9/433/s1. Table S1: Number of mapped reads per million reads of 22 LTR-RE families in eightdomesticated genotypes and seven wild accessions, Table S2: Number of gene-RE mapping paired reads per million reads of 22 LTR-RE families in eight domesticated genotypes and seven wild accessions, Table S3: List of genes mapped by gene-RE paired Illumina reads of domesticated (a) and wild (b) genotypes.
